# Combination of *Spirulina platensis*, *Ganoderma lucidum* and *Moringa oleifera* Improves Cardiac Functions and Reduces Pro-Inflammatory Biomarkers in Preclinical Models of Short-Term Doxorubicin-Mediated Cardiotoxicity: New Frontiers in Cardioncology?

**DOI:** 10.3390/jcdd9120423

**Published:** 2022-11-28

**Authors:** Vincenzo Quagliariello, Manuela Giovanna Basilicata, Giacomo Pepe, Raffaele De Anseris, Annabella Di Mauro, Giosuè Scognamiglio, Giuseppe Palma, Vincenzo Vestuto, Simona Buccolo, Antonio Luciano, Massimiliano Barbieri, Francesca Bruzzese, Carlo Maurea, Rossella Pumpo, Carmine Ostacolo, Pietro Campiglia, Massimiliano Berretta, Nicola Maurea

**Affiliations:** 1 Division of Cardiology, Istituto Nazionale Tumori-IRCCS-Fondazione G. Pascale, 80131 Naples, Italy; 2 Department of Pharmacy, University of Salerno, 84084 Fisciano, Italy; 3 Anseris Pharma, 83029 Avellino (AV), Italy; 4 Pathology Unit, Istituto Nazionale Tumori-IRCCS-Fondazione G. Pascale, 80131 Naples, Italy; 5 Department of Precision Medicine, University of Campania “L. Vanvitelli”, 80131 Naples, Italy; 6 Animal Facility, Istituto Nazionale Tumori-IRCCS-Fondazione G. Pascale, 80131 Naples, Italy; 7 Department of Neurology, University of Salerno, 84084 Fisciano, Italy; 8 Digestive Endoscopy Unit S. G. Bosco Hospital, ASLNA1, 80144 Naples, Italy; 9 Department of Pharmacy, University of Naples Federico II, 80138 Naples, Italy; 10 Department of Clinical and Experimental Medicine, University of Messina, 98122 Messina, Italy

**Keywords:** cardiotoxicity, cardioprotection, nutraceuticals, inflammation, strain, cancer

## Abstract

Anthracyclines are essential adjuvant therapies for a variety of cancers, particularly breast, gastric and esophageal cancers. Whilst prolonging cancer-related survival, these agents can induce drug-related cardiotoxicity. Spirulina, Reishi (*Ganoderma lucidum*) and Moringa are three nutraceuticals with anti-inflammatory effects that are currently used in cancer patients as complementary and alternative medicines to improve quality of life and fatigue. We hypothesize that the nutraceutical combination of Spirulina, Reishi and Moringa (Singo) could reduce inflammation and cardiotoxicity induced by anthracyclines. Female C57Bl/6 mice were untreated (Sham, n = 6) or treated for 7 days with short-term doxorubicin (DOXO, n = 6) or Singo (Singo, n = 6), or pre-treated with Singo for 3 days and associated with DOXO for remaining 7 days (DOXO–Singo, n = 6). The ejection fraction and radial and longitudinal strain were analyzed through transthoracic echocardiography (Vevo 2100, Fujifilm, Tokyo, Japan). The myocardial expressions of NLRP3, DAMPs (galectin-3 and calgranulin S100) and 13 cytokines were quantified through selective mouse ELISA methods. Myocardial fibrosis, necrosis and hypertrophy were analyzed through immunohistochemistry (IHC). Human cardiomyocytes were exposed to DOXO (200 nM) alone or in combination with Singo (at 10, 25 and 50 µg/mL) for 24 and 48 h. Cell viability and inflammation studies were also performed. In preclinical models, Singo significantly improved ejection fraction and fractional shortening. Reduced expressions of myocardial NLRP3 and NF-kB levels in cardiac tissues were seen in DOXO–Singo mice vs. DOXO (*p* < 0.05). The myocardial levels of calgranulin S100 and galectin-3 were strongly reduced in DOXO–Singo mice vs. DOXO (*p* < 0.05). Immunohistochemistry analysis indicates that Singo reduces fibrosis and hypertrophy in the myocardial tissues of mice during exposure to DOXO. In conclusion, in the preclinical model of DOXO-induced cardiotoxicity, Singo is able to improve cardiac function and reduce biomarkers involved in heart failure and fibrosis.

## 1. Introduction

Doxorubicin (DOXO) is a chemotherapeutic agent prescribed to treat several types of cancers [1]. This anti-cancer drug has various side effects, such as allergic reactions, cardiac damage, hair loss, bone marrow suppression, vomiting, and bladder irritation [2]. The most dangerous side effect of DOXO is cardiomyopathy, leading to congestive heart failure [3]. The mechanisms of DOXO-induced cardiotoxicity are mediated by the overexpression of interleukins, inflammasome and ferroptosis [4,5]. DOXO-induced cardiotoxicity starts from myocardial cell damage, inducing DAMPs production and cell ferroptosis that induce left ventricular dysfunctions [6]. Many factors and multiple pathways are responsible for the creation of DOXO-induced cardiotoxicity: inflammatory cytokines, oxidative stress pathways, mitochondrial damage, intracellular Ca^2+^ overload, iron-free radical production, DNA and myocyte membrane injuries have critical roles in the pathophysiology of DOXO-induced cardiotoxicity [7,8]. Unfortunately, there are currently no cardioprotective agents able to offer a complete spectrum of the primary and secondary prevention of DOXO-induced cardiomyopathies [9]. Recent preclinical and clinical studies focused on SGLT-2 inhibitors [10], PCSK9 inhibitors [11], sacubitril-valsartan and selective cytokine inhibitors [12] have demonstrated some beneficial cardiorenal effects in non-cancer and cancer patients treated with cardiotoxic drugs. Nutraceuticals are a group of naturally produced bioactive compounds that have proven health benefits besides their nutritive properties [13,14,15]. In this specific cluster, microalgae have stood out as photosynthetic microorganisms capable of generating biofunctional molecules with several cytoprotective activities. Particularly, spirulina is a filamentous cyanobacterium that accounts for up to 30% of the overall microalgal biomass produced worldwide [16]. Spirulina is primarily composed of proteins and essential amino acids providing high nutritional value but additionally contains phenolic phytochemicals including C-phycocyanin, vitamins, polyunsaturated fatty acids and an elevated concentration of beta-carotene, delivering substantial antioxidative, anti-inflammatory and anti-atherosclerotic properties beyond its beneficial effects in metabolic-associated cardiovascular disorders [17,18]. A systematic review including 18 randomized controlled trials has reported that spirulina supplementation is safe and displays positive effects in multiple metabolic syndrome components [19]. As such, spirulina has been shown to exert a noteworthy weight management capacity, eliciting a reduction in both waist circumference and body mass index in several clinical trials [20,21]. Moreover, several studies with spirulina have shown an improvement in insulin sensitivity and glucose uptake mediated by C-phycocyanin activity, and, in some studies, spirulina has displayed hypolipidemic properties [22,23,24]. On the other hand, cell culture approaches have demonstrated the ability of spirulina to exhibit anti-atherosclerotic effects by preventing monocyte migration through the direct inhibition of P- and E- selectin adhesion molecules [25,26] and to effectively inhibit DOXO-induced cardiac damage [27,28]

The Moringa genus is a subtropical tree native to Asia and Africa, which includes 13 species; *Moringa oleifera Lam*. (MO) is the most cultivated for its beneficial uses. MO is also known as the “miracle tree” because it has been used traditionally as a food source and medicine to treat various diseases such as anemia, diabetes and infectious or cardiovascular diseases [29]. The phytochemical compounds identified in MO with functional activities associated with cardiovascular diseases are N,α-L-rhamnopyranosyl vincosamide, isoquercetin, quercetin, quercetin and isothiocyanate [30,31].

*Ganoderma lucidum*, which is known in Chinese as “Lingzhi”, or Reishi, is a medicinal mushroom commonly used as a Chinese herbal medicine and the main ingredient in many conventional combinations or dietary supplements [32]. Reishi exerts several beneficial effects, including immunomodulation, anticancer and anti-inflammatory activity [33,34]. The active constituents include polysaccharides and oxygenated triterpenoids, which have a broad range of biological activities and pharmacological functions. In vitro studies with certain extracts have shown effects that may benefit the cardiovascular system including the inhibition of cholesterol synthesis, the lowering of blood pressure by decreasing sympathetic outflow from the central nervous system and antioxidant effects [35]. We aimed to assess if the oral administration of a nutraceutical combination of Reishi, Spirulina and Moringa (a complex called Singo, at a molar ratio of 2:1:1 between the molecules, respectively) could improve cardiac function and reduce pro-inflammatory biomarkers in preclinical models of anthracycline-induced cardiotoxicity [36].

## 2. Materials and Methods

### 2.1. Protein Digestion of Spirulina platensis and Moringa oleifera Extracts

#### 2.1.1. Protein Content Determination

The protein content determination of *Spirulina platensis* and *Moringa oleifera* aqueous extracts was carried out using the Bradford method [37]. Comassein Blue G-250 was used as a protein binding dye, and the sample was spectrophotometry monitored at 595 nm. Bovine serum albumin (BSA) was used as a standard protein to draw a calibration curve and find the protein concentration (y = 887.17241x − 23.21075; R^2^ > 0.99).

#### 2.1.2. Derivatization and Digestion

After the protein content determination, trypsin digestion was carried out in solution. Briefly, the lyophilized extracts were solubilized in 6 M Urea and 50 mM Tris-HCl at pH 8.0. Subsequently, 5 µL of 200 mM DTT dithiotreitol (50 mM Tris-HCl at pH 8.0) was added followed by incubation for 60 min at 25 °C in a Thermomixer comfort (Eppendorf, Hamburg, Germany). Reduced cysteine amino acids were alkylated by adding 20 µL of 200 mM Iodoacetamide (50 mM Tris-HCl at pH 8.0). The mixture was gentle vortex and incubated for 1 h at room temperature in dark. Then, 20 µL of 200 mM DTT was added to consume any unreacted iodoacetamide. Trypsin solution to a final ratio of 1:50 (*w*/*w*, trypsin:protein) was added and incubated overnight at 37 °C. Finally, trypsin activity was inhibited by acidification with 3 µL of formic acid (FA) solution. Samples were centrifuged for 10 min at 14,680 rpm (Eppendorf microcentrifuge 5424) and the supernatants were dried under vacuum for 2 h at 35 °C (Savant SPD140DDA SpeedVac Concentrator connected to an RVT5105 Refrigerated Vapor Trap, Thermo Scientific, Winsford, UK) [38].

#### 2.1.3. Desalting

To remove the buffer salts, tryptic digests were purified using C18 Stagetips (Thermo Scientific). Briefly, C18 Stagetips were wetted with MeOH and then 80% ACN/0.1% TFA and equilibrated with 80% ACN/0.25% TFA. In total, 20 µL of each solution was added sequentially to the top and centrifuged for 2 min at 2000 rpm at 25 °C. After loading the sample, the C18 Stagetips were washed three times with 20 µL 0.1% TFA, and peptide digest was eluted in a low protein binding tube (Thermo Scientific) with H_2_O 0.1% TFA. The eluate was evaporated in a SpeedVac and reconstituted with 0.1% FA to a total volume of 50 µL and analyzed by NanoLC-HRMS.

#### 2.1.4. Peptide Separation

A nanoflow ultra-high-performance liquid chromatography (UHPLC) instrument (Ultimate 3000, Thermo Fisher Scientific, Bremen, Germany) was coupled online to an Orbitrap Fusion™ Lumos™ Tribrid Mass Spectrometer (Thermo Scientific) fitted with the Nanospray Flex NG ion source (Thermo Fisher Scientific). The peptides were trapped on a PepMap trap column (Thermo Fisher) for 2.0 min at a flow rate of 40 µL min^−1^, and then the peptides were loaded and separated onto a C18 reversed-phase nano column (0.075 ID × 250 mm × 2.6 µm biozenTM Peptide XB-C18, Phenomenex, Bologna, Italy). The mobile phases were A: 0.1% FA in H_2_O; B: 0.1%FA in ACN/H_2_O 80/20. A linear gradient to 35% buffer B over 130 min was used to elute the majority of the peptides before increasing buffer B to 90% over 20 min and holding for an additional 10 min at a flow rate of 300 nL min^−1^. The column was re-equilibrated for 10 min with 4% buffer B before the next injection.

### 2.2. Mass Spectrometry and Protein Analysis

The eluted peptides were ionized for detection by the Orbitrap Fusion™ Lumos™ Tribrid Mass Spectrometer (Lumos) operating in positive polarity. The source conditions were as follows: voltage, 2.0 kV, transfer tube temperature, 305 °C. Peptides (MS1) were detected in the Orbitrap with the following settings: resolving power 120,000 at *m*/*z* 200; scan range *m*/*z* 300–1500; S-Lens RF 30%; microscans, 1; ACG values were set to auto. The top 15 peptides were selected in the data-dependent mode for MS/MS fragmentation by CID at 35% NCE to be detected by ion trap with dynamic exclusion for 60 s.

The raw MS data were analyzed using Protein Discoverer™ Software version 2.5 using the Sequest search engine. The spectra were searched against the *Arthrospira platensis* and *Arabidopsis thaliana* databases from UniprotKB [39,40]. Precursor mass tolerance was set to 10 ppm. Enzyme specificity was set to trypsin, and two missed cleavages were allowed. Carbamidomethyl cysteine was set as a fixed modification, methionine oxidation, asparagine and glutamate deamidation, and the phosphorylation of serine, threonine and tyrosine were set as variable modifications.

### 2.3. Determination of Total Polysaccharides Content in Aqueous Extract of G. Lucidum

The total polysaccharide content of the aqueous extract was determined using the phenol-sulfuric acid method [41]. Briefly, 1 mL of 5% (*w*/*v*) phenol was added to 1 mL of sample solution and 5 mL of concentrated sulfuric acid. After allowing the test tubes to stand for 10 min, they are vortexed for 30 s and placed for 20 min in a water bath at 30 °C for color development. The absorbance was measured using a spectrophotometer UV/Vis (Evolution 201, Thermo Fisher Scientific) at 490 nm. Reference solutions are prepared in an identical manner as above, except that the sample is replaced by double-distilled water. D-glucose was selected as the standard. Stock solution (10 mg mL^−1^) was prepared in water, and the calibration curve was obtained in a concentration range of 20–800 mg L^−1^, with six concentration levels (y = 208.48x − 24.73). The solution was measured in triplicate.

### 2.4. Polysaccharide Extraction and Fourier Transform Infrared (FT-IR) Characterization

The polysaccharides were isolated from *G. lucidum* aqueous extract following a protocol previously described by Kan et al. (2015) applying the following experimental conditions: ratio of water to material: 30 mL g^−1^; extraction time and temperature: 120 min and 60 °C, respectively [42]. After the extraction was completed, cooling, centrifugal separation (6000 rpm, 10 min, 4 °C), and filtering were carried out in sequence to obtain a supernatant fluid containing *G. lucidum* polysaccharides (GLP). Finally, the GLPs were lyophilized for 24 h (LyoQuest-55, Telstar Technologies, Terrassa, Spain) using the condenser temperature of −52 °C and 0.100 mBar as the vacuum value. Dry GLP extract was analyzed at 4000–600 cm^−1^ at 25 °C using a Spotlight 400 FT-IR Imaging System (PerkinElmer, Waltham, MA, USA) to determine the organic functional groups of GLPs.

### 2.5. Animal Studies

**Figure 1:**
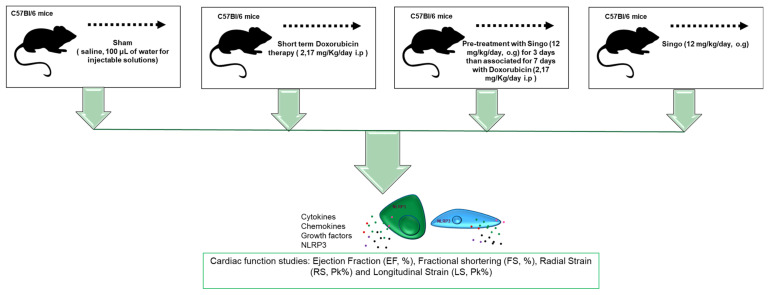
Overall design of the preclinical study of DOXO-induced cardiotoxicity and cardioprotective strategy based on oral gavage of Singo, a nutraceutical combination of Reishi, Spirulina and Moringa at 12 mg/kg.

Twenty-four female C57Bl/6 mice were purchased from Envigo Laboratories, Indianapolis, IN, USA. The mice were housed (5 per cage) and maintained on a 12 h light–12 h dark cycle (lights on at 7.00 am) in a temperature-controlled room (22 ± 2 °C) with food and water ad libitum. The experimental protocols, in accordance with the EU Directive 2010/63/EU for animal experiments and Italian D.L.vo 26/2014 law, were approved by the Ministry of Health with authorization number 1467/17-PR of 13-02-2017, and institutional ethics committees: Organismo preposto al benessere degli animali (OPBA). The mice were randomized for weight in treatment groups (n = 6) considering an average of 20.5 g per group (Figure 1): Sham/control (n = 6): 10 days i.p. saline (100 μL of water for injectable solutions), Doxorubicin (DOXO) (n = 6): 3 days i.p. saline, then 7 days i.p. DOXO at 2.17 mg/kg/day, Singo (n = 6): 10 days of oral Singo (combination of Spirulina, Reishi and Moringa) at 12 mg/kg); Singo + DOXO (Singo–DOXO) (n = 6): 3 days of oral Singo, then 7 days of oral Singo + i.p. DOXO (Figure 1). The cardioprotective regimen was based on the same preclinical protocol followed by our research group [43].

#### 2.5.1. Echocardiographic Evaluation of Ventricular Functions

To assess cardiac functions, non-invasive transthoracic echocardiography was performed in sedated mice through a Vevo 2100 high-resolution imaging system (40-MHz transducer; Visual Sonics, Toronto, ON, Canada) as described in the literature [36,43]. In brief, the mice were anesthetized with tiletamine (0.09 mg/g), zolazepam (0.09 mg/g) and 0.01% atropine (0.04 mL/g). Once sedated and placed in the supine position on a temperature-controlled surgical table to maintain the rectal temperature at 37 °C, continual ECG monitoring was obtained via limb electrodes. The cardiac function was evaluated by echocardiography in basal conditions and once per week for the three weeks of treatment. The LV echocardiography was assessed in parasternal long-axis views at a frame rate of 233 Hz. The end-systole and end-diastole dimensions were defined as the phases corresponding to the ECG T wave and to the R wave, respectively. M-mode LV internal dimensions and diastolic (LVID,d) and systolic (LVID,s) LV internal dimensions were averaged from 3 to 5 beats. The LVID,d and LVID,s were measured from the LV M-mode at the mid-papillary muscle level. The fractional shortening percentage (% FS) was calculated as [(LVID, d-LVID, s)/LVID, d] × 100, and the ejection fraction percentage (% EF) was calculated as [(EDvol-ESvol)/EDvol] × 100. The analysis started with acquired B-mode loops which were imported into the Vevo Strain software. Three consecutive cardiac cycles were selected, and the endocardium was traced. Upon the adequate tracing of the endocardium, an epicardial trace was added. The ST-based strain allowed the assessment of strains specific to 6 myocardial segments per LV view. Internally, 10 or more points were measured for each of the 6 segments, resulting in 48 data points in total. The strain was evaluated on long-axis views as well as radial and longitudinal. Radial strain (RS), defined as the percent change in myocardial wall thickness, is reported as a positive curve reflecting increasing myocardial thickness during systole and diminishing wall thickness during diastole, representing myocardial deformation toward the center of the LV cavity. Longitudinal strain (LS) detects the percent change in the length of the ventricle, typically measured from the endocardial wall in the long-axis view [44,45]. The values will be reported as the mean values of 6 animals per group.

#### 2.5.2. Cardiac Inflammation and DAMPs

After the treatments, the mice were sacrificed by cervical dislocation after anesthesia with tiletamine (0.09 mg/g), zolazepam (0.09 mg/g) and 0.01% atropine (0.04 mL/g), and the total heart was weighed and processed for inflammation studies. The heart tissues were snap-frozen using dry ice until later use for tissue homogenization, which was carried out in 0.1 M phosphate-buffered saline (pH 7.4) containing 1% TritonX-100 and protease inhibitor cocktail, and which was processed by using a high-intensity ultrasonic liquid processor. The homogenates were centrifuged at 4 °C, and the supernatants were used for the quantification of multiple inflammation markers, as described: NOD-, LRR- and pyrin domain-containing protein 3 (NLRP3) inflammasome expression (ng/mL of tissue extract) was quantified by an NLRP3 ELISA Kit (Mouse) (OKEH05486) (Aviva Systems Biology). Moreover, 12 cytokines involved in inflammation (IL-1α, IL-1β, IL-2, IL-4, IL-6, IL-10, IL-12, IL-17α, IFN-γ, TNF-α, G-CSF, GM-CSF) were quantified in the heart tissue extracts by using the 12 mouse cytokine Multiplex Assay kit (Qiagen, Redwood City, CA, USA) following the manufacturer’s instructions; the results were expressed as the pg of the cytokine/mg of heart tissue [46]. DAMPs (S100/Calgranulin and Galectin-3) were quantified in the cardiac tissues through a Mouse S100 Calcium Binding Protein A9 (S100A9) Calgranulin ELISA Kit (MyBioSource, Milan, Italy) and a Mouse Galectin 3 ELISA Kit (ab203369) (AbCam, Milan, Italy), respectively. The values will be reported as the mean values of 6 animals per group.

#### 2.5.3. Haematoxylin and Eosin (H&E) and Masson’s Trichrome Staining in Myocardial Tissues

Blinded histological examinations of myocardial tissues were also performed. All selected samples were fixed in formalin and embedded in paraffin. Firstly, the tissues were deparaffinized in a solution of xylene and rehydrated through graded alcohols. To determine the structure of the heart, and to evaluate parameters such as hypertrophy, necrosis and fibrosis, the tissues of the heart were incubated with Mayer’s hematoxylin for 30 s and washed with tap water [47]. Masson’s trichrome staining for the collagen level assessment was also performed. The interstitial fibrotic areas were evaluated with image analysis software (Image-pro plus 6.0; Media Cybernetics LP, Washington, DC, USA).

#### 2.5.4. Terminal dUTP Nick End-Labelling (TUNEL) Assay

The heart samples were fixed with 10% formaldehyde, paraffin embedded and cut into 4 μm-thick sections. A DeadEnd™ Fluorometric TUNEL System was used to assess apoptosis in the mouse heart samples. A TUNEL panel was performed for the fluorescence staining of the fragmented DNA in the apoptotic cells. Images were recorded digitally using an LMD camera (Leica) fitted with 20× and 40× objectives, and the samples were analyzed by counting the positively stained nuclei.

### 2.6. Cell Studies: Cell Viability

To evaluate the cytotoxic or cytoprotective effects of Singo, the mitochondrial dehydrogenase activity was quantified through a modified MTT [3-(4,5-dimethyldiazol-2-yl)-2,5-diphenyl tetrazolium bromide] method, called MTS assay, according to the manufacturer’s instructions (Dojindo Molecular Technologies Inc., Rockville, MD, USA) [48]. Briefly, human fetal cardiomyocytes (HFC cells, Innoprot, Derio, Spain) were cultured in Cardiac Myocyte Medium (CMM, Innoprot, Derio—Bizkaia, Spain), added to 2 mM l-glutamine, 100 U/mL penicillin and 100 μg/mL streptomycin in 96-well plates (density of 10,000 cells/well) at 37 °C in a humidified 5% CO_2_ atmosphere according to the manufacturer’s recommendations. After 24 h of appropriate growth, the cells were exposed for 24 and 48 h to: DOXO (200 nM); Singo (10, 25 and 50 µg/mL); DOXO–Singo (cells co-treated with Singo and DOXO). After the treatments, the cells were washed three times with phosphate-buffered solution (PBS) at pH 7.4 and then incubated with 100 μL of an MTS solution (0.5 mg/mL in cell culture medium) for 4 h at 37 °C. Absorbance readings were acquired at a wavelength of 450 nm with the Tecan Infinite M200 plate reader (Tecan Life Sciences Home, Männedorf, Switzerland) using I-control software (Tecan). The relative cell viability (%) was calculated with the following formula [A]test/[A]control  ×  100, where “[A]test” is the absorbance of the test sample and “[A]control” is the absorbance of the control cells incubated solely in culture medium.

#### 2.6.1. Lipid Peroxidation

To study the putative antioxidant effects of Singo, cardiomyocytes were grown as described above. Subsequently, 5 × 10^3^ cells/well were seeded in a 24-well plate and allowed to grow for 24 h and exposed to DOXO (200 nM) or pre-treated for 4 h with Singo (10, 25 and 50 µg/mL). After centrifugation at 800× *g* for 5 min, the supernatant was evaluated for malondialdehyde (MDA) (Lipid Peroxidation (MDA) Assay Kit) and 4-hydroxy 2-hexenal (4-HNA) (Universal 4-Hydroxynonenal ELISA Kit, NBP2-66364) using commercial kits with a spectrophotometer according to the manufacturer’s protocols (Sigma Aldrich, Milan, Italy, and Bio-Techne SRL, Milan, Italy, respectively) [49].

#### 2.6.2. p65/NF-kB Expression

The cardiomyocytes were treated with DOXO (200 nM), Singo (10, 25 and 50 µg/mL) or DOXO and Singo for 24 h. Afterwards, the nuclear extracts were analyzed using the TransAM p65/NF-κB transcription factor assay kit (Active Motif, Carlsbad, CA, USA) according to the manufacturer’s recommendations. The data were expressed as the percentage of p65/NF-kB DNA binding versus control (untreated) cells [50].

#### 2.6.3. NLRP3 Expression

The cardiomyocytes were treated with DOXO (200 nM), Singo (10, 25 and 50 µg/mL) or DOXO and Singo for 24 h. After the treatments, the cells were harvested and lysed in complete lysis buffer (50 mM Tris–HCl, pH 7.4, 1 mM EDTA, 100 mM NaCl, 20 mM NaF, 3 mM Na_3_VO_4_, 1 mM PMSF and protease inhibitor cocktail). After centrifugation, supernatants were collected and treated to the quantification of NLRP-3 (human NLRP-3 ELISA Kit, Aviva Systems Biology. The sensitivity of the human NLRP-3 ELISA was above 0.078 ng/mL, and the range of detection was 0.156–10 ng/mL [51].

#### 2.6.4. Intracellular Cytokines

The cardiomyocytes were treated with DOXO (200 nM), Singo (10, 25 and 50 µg/mL) or DOXO and Singo for 24 h. After the treatments, the cells were harvested and lysed in complete lysis buffer (50 mM Tris–HCl, pH 7.4, 1 mM EDTA, 100 mM NaCl, 20 mM NaF, 3 mM Na_3_VO_4_, 1 mM PMSF and protease inhibitor cocktail). After centrifugation, the supernatants were collected and analyzed for cytokines (IL-1α, IL-1β, IL-2, IL-4, IL-6, IL-10, IL-12, IL-17α, IFN-γ, TNF-α, G-CSF, GM-CSF) through a 12-human cytokine Multiplex Assay kit (Qiagen, USA) following the manufacturer’s instructions; the results were expressed as the pg of cytokine/mL [52].

### 2.7. Statistics

The data are presented as means ± standard deviation (SD). Analysis of variance (ANOVA) with Sidak correction for multiple comparisons was applied to compare the different groups. Values of *p* < 0.05 were considered statistically significant. In each figure, the *p*-value of the ANOVA is shown along with symbols for the individual comparisons between specific groups.

## 3. Results

### 3.1. Chemical and Physical Analysis of Plant Extract

Firstly, we investigated the nutraceutical potential of *Spirulina platensis*, *Moringa oleifera* and *Ganoderma lucidum* extracts (the chemical components of Singo).

**Figure 2:**
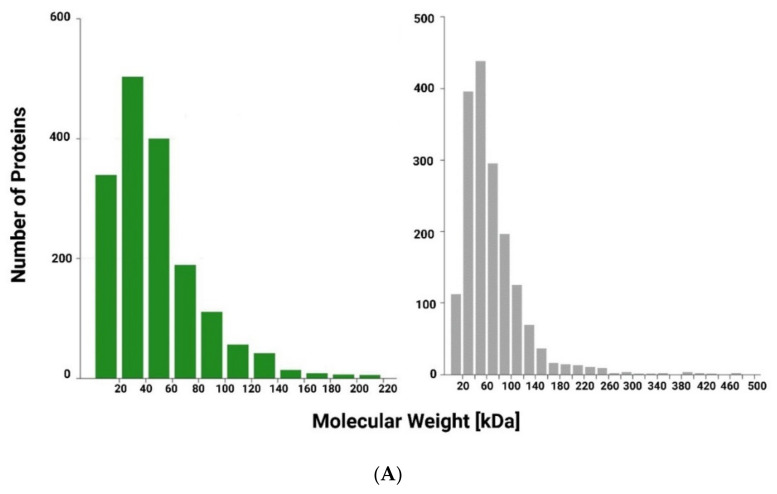

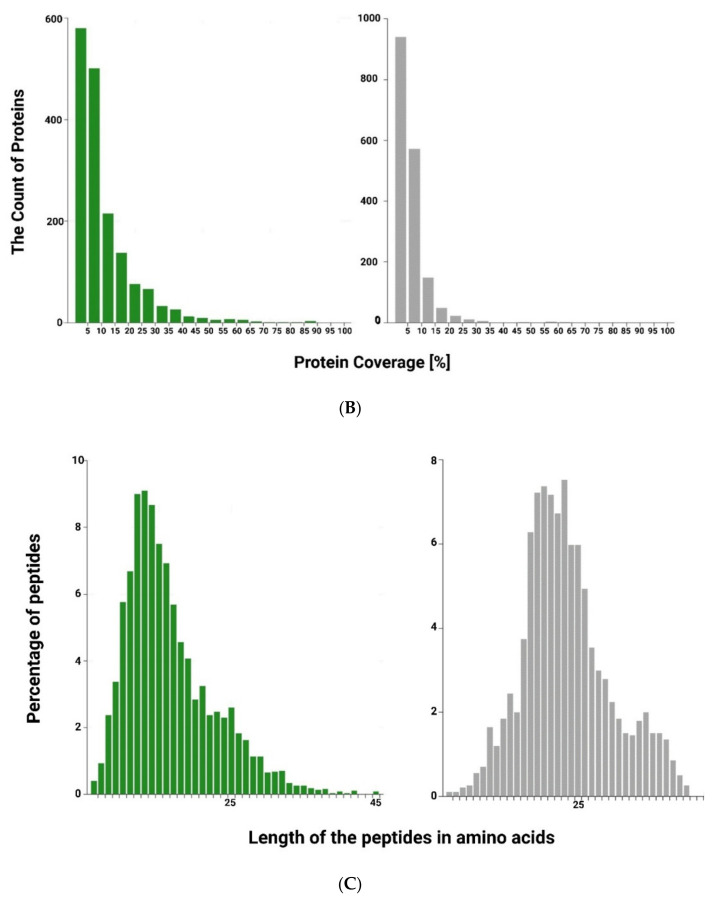
(**A**) Distribution of relative molecular mass for all identified proteins in *Arthrospira platensis* (green) and in *Moringa oleifera* (grey) extracts; (**B**) Distribution of protein sequence coverage for all identified proteins; (**C**) Distribution of lengths for all identified peptides.

*Spirulina platensis* and *Moringa oleifera* are important protein sources due to their high nutritional value protein contents, as confirmed by Bradford assay [39] (protein content > 60% *w*/*w*). To determine the protein composition of the *Arthrospira platensis* and *Moringa oleifera* extracts, a label-free based liquid chromatography–mass spectrometry (LC–MS/MS) proteomic approach was carried out. Figure 2 shows the distribution of the molecular weight (A), protein sequence coverage (B) and the profile of lengths (C) for all identified peptides in the *Arthrospira platensis* and *Moringa oleifera* extracts (Figure 2). The complete list of proteins identified is reported in the Supplementary Materials.

The polysaccharide content of the *Ganoderma lucidum* extract was determined by the phenolic–sulfuric acid method. In this assay, the concentrated sulfuric acid broke down all polysaccharides into monosaccharides. The simple sugars thus formed were subsequently dehydrated to furfural or hydroxymethyl furfural. These compounds reacted with phenol, resulting in an orange-gold hue.

**Figure 3:**
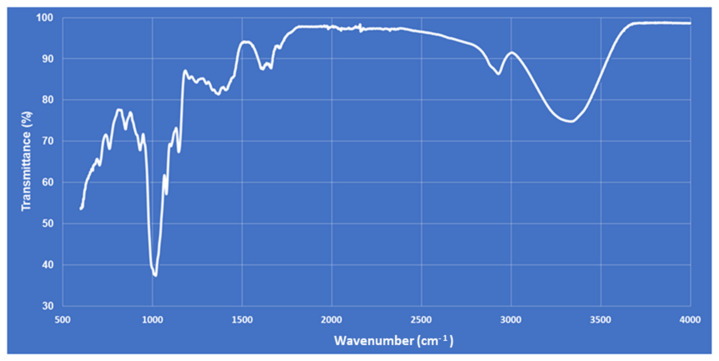
FT-IR spectra of GLP.

The validation of the spectrophotometric method was carried out using standard D-glucose, the maximum absorbance of the D-glucose was recorded at 490 nm and the linearity of the standard curve was 99.82%. The polysaccharide content in the *Ganoderma lucidum* extract was 32.5 ± 0.1% (*w*/*w*). The characteristic functional groups of GLPs were identified by FT-IR spectroscopy. The FT-IR spectrum was reported in the range of 4000–500 cm^−1^ (Figure 3) and showed the characteristic absorbance at about 3341.5 cm^−1^, 2934.5 cm^−1^ and 1662.5 cm^−1^ for the –OH groups and the C–H and C = O bonds, respectively (Figure 3). The spectrum at 990–1050 cm^−1^ confirmed the presence of β-glycosidic linkage.

### 3.2. Cardiac Function Studies

**Figure 4:**
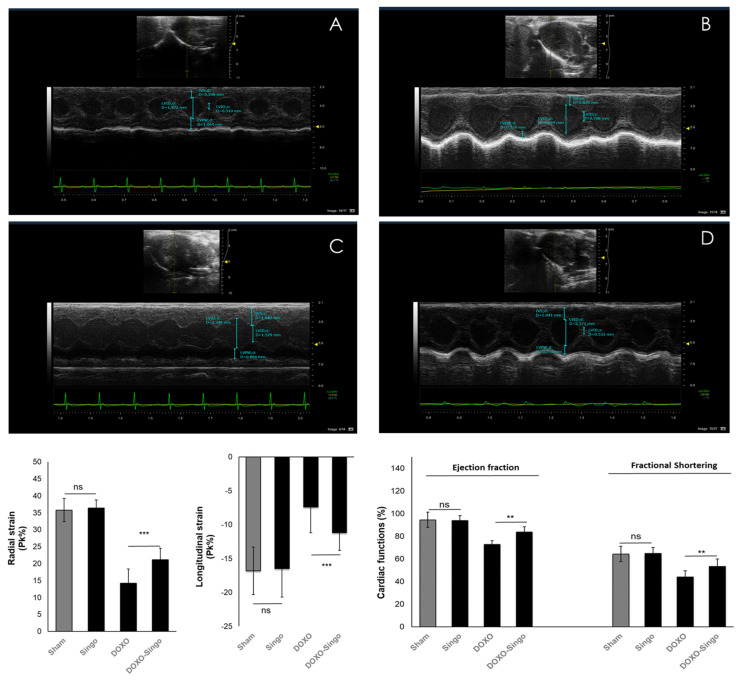
Cardiac function studies in mice untreated (saline) or treated with Singo at 12 mg/kg/day, Doxorubicin (DOXO) at 1.25 mg/kg/day and both in combination for 10 days. Vevo 20,100 model was used as echocardiography for the determination of radial strain, longitudinal strain, ejection fraction and fractional shortening. Up: A characteristic picture of cardiac contractility in mice untreated (**A**), treated with Singo (**B**) or DOXO (**C**) or both in combination (**D**). Down: Radial strain (Pk %), Longitudinal Strain (Pk %), Ejection Fraction (%) and Fractional Shortering (%) in mice untreated or treated with Singo or DOXO or both in combination. Results: mean +/− SD. ns: not significant; *** *p* < 0.001. ** *p* < 0.01.

Short-term treatment with DOXO was associated with a significant reduction in EF (−10.3% compared to baseline: *p*  <  0.05) and FS (−19.8% compared to baseline; *p*  <  0.05). Singo did not change the cardiac functions in the mice compared to the untreated ones (Figure 4). The DOXO–Singo mice had a significant improvement in EF (88.3  ±  2.3 vs. 81.2  ±  2.5 (%); *p*  <  0.05) and FS (58.7  ±  3.5 vs. 48.8  ±  2.9 (%); *p*  <  0.05) compared to the DOXO group (Figure 4). Strain on long-axis images and ventricular functions are defined as useful echocardiographic markers of cardiotoxicity [53]. The strain anabudder showed that Singo significantly improves cardiac functions when used in combination with DOXO compared to DOXO-treated mice (quantitative data in Figure 4, down). In particular, the radial strain (RS) is 30.3% in Singo–DOXO vs. 15.7% in DOXO mice (*p*  <  0.001); longitudinal strain (LS) is −17% in Singo–DOXO vs. −11.7% in DOXO mice (*p*  <  0.001) (Figure 4).

### 3.3. Myocardial DAMPs and Inflammasome Expression

**Figure 5:**
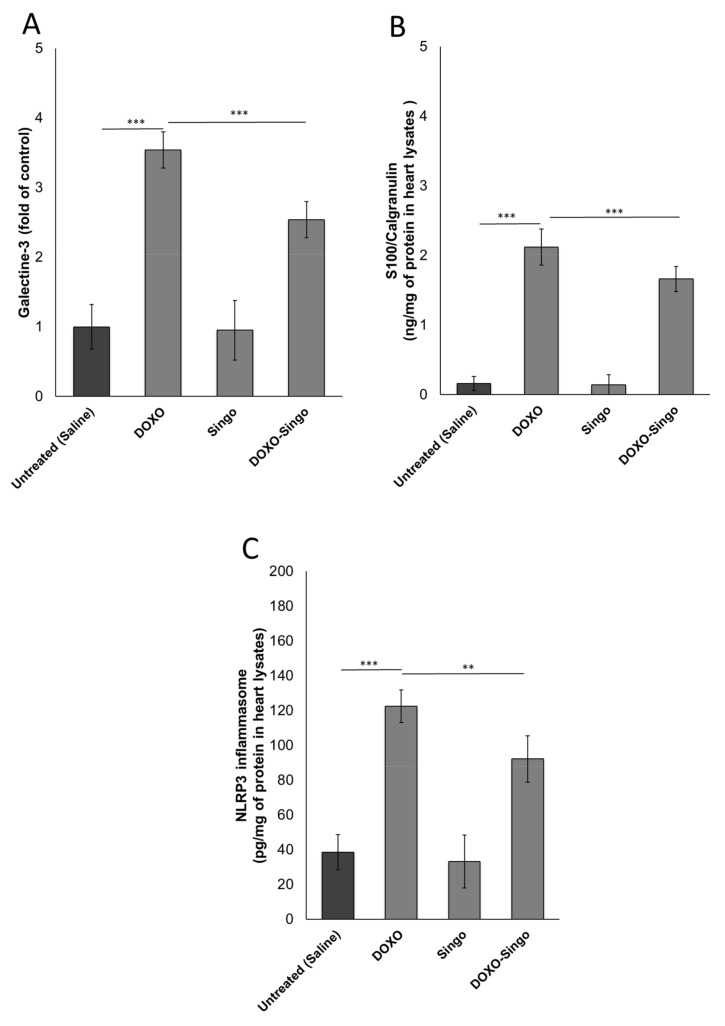
Cardiac DAMPs (Galectin-3 and S-100 calgranulin) (**A**,**B**) and NLRP-3 (**C**) expression in the myocardial tissue of mice untreated (saline) or treated with DOXO, Singo or both in combination for 10 days. Results: mean +/− SD. *p*-values: *** *p* < 0.001. ** *p* < 0.01.

DAMPs are proteins involved in cell damage induced by viruses, bacteria, cancer and also cardiotoxic drugs [54]. Galectin-3 and S100/Calgranulin high plasma levels are associated with heart failure, cardiac fibrosis and atherosclerosis [55]. Mice treated with DOXO had high myocardial levels of DAMPs compared to the saline group (*p* < 0.001). In contrast, co-treatment with Singo reduces significantly both DAMPs compared to DOXO mice (*p* < 0.001 vs. DOXO) (Figure 5). Similarly, NLRP-3 inflammasome expression in myocardial tissue was significantly reduced in DOXO–Singo vs. DOXO-treated mice (*p* < 0.001) (Figure 5).

### 3.4. Cytokinome Profile Evaluation in Myocardial Tissue

**Figure 6:**
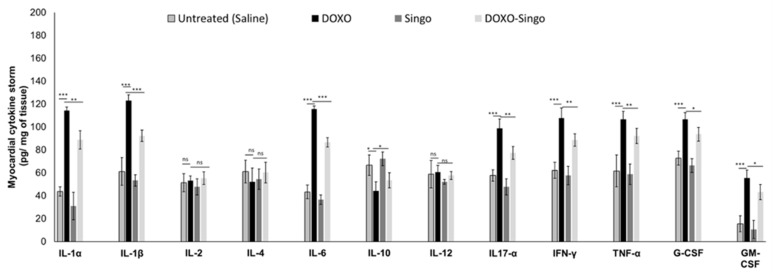
Myocardial cytokines, chemokines and growth factors in preclinical models of DOXO-induced cardiotoxicity (IL-1α, IL-1β, IL-2, IL-4, IL-6, IL-10, IL-12, IL-17α, IL-18, IFN-γ, TNF-α, G-CSF and GM-CSF). Results: mean +/− SD. *p*-values for the indicated compounds relative to untreated mice are: ns: not significant. *** *p* < 0.001. ** *p* < 0.01. * *p* < 0.05.

A pro-inflammatory cytokine profile was associated with cardiomyopathies induced by anticancer drugs or immune checkpoint inhibitors [56]. Here, changes in cytokine levels in the myocardial tissue of mice treated with DOXO were analyzed. As indicated in the literature, short-term DOXO therapy increases IL-1α and β, IL-6. IL-17α and growth factors in the myocardial tissue of preclinical models as well as in cancer patients. Nutraceutical oral administration did not significantly change all cytokines and chemokines analyzed (Figure 6). In contrast, in DOXO–Singo mice, IL-1α/β, IL-6, IL-17α and TNF-α levels were significantly reduced compared to the DOXO group (*p* < 0.005). In contrast, the IL-10 levels were increased in the heart of DOXO–Singo mice, indicating anti-inflammatory effects (Figure 6).

### 3.5. Histology Evaluations: H&E and Masson’s Trichrome Staining in Myocardial Tissues

**Figure 7:**
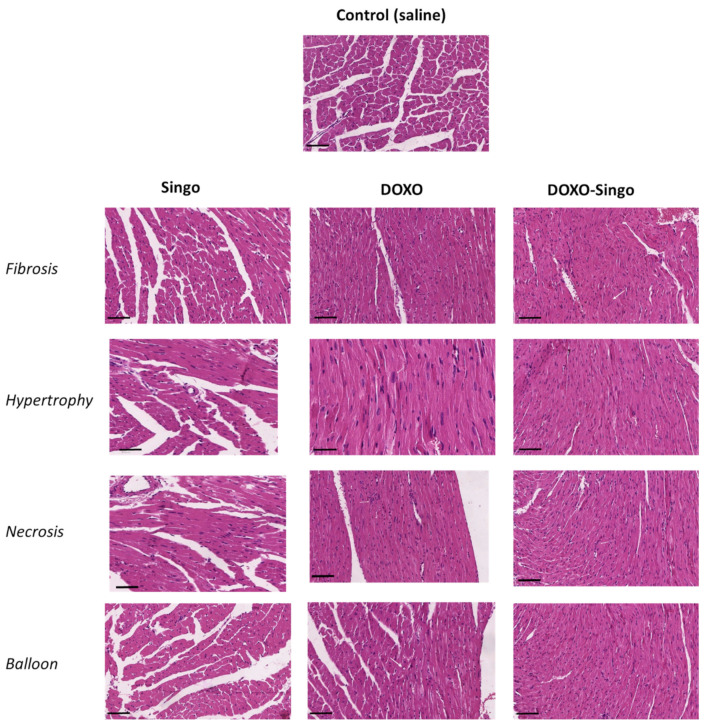
Histological study of cardiac tissues of mice not treated or treated with DOXO, Singo or both in combination. At the end of the experiment, after appropriate sacrifice, the tissues were analyzed for the evaluation of hypertrophy, fibrosis, necrosis and balloon. Scale bar: 50 µm.

Histological investigations were performed in order to evaluate structural changes in the myocardium in mouse models treated with short-term DOXO therapy alone or in combination with the Singo nutraceutical (Figure 7). Anthracyclines induce many structural and metabolic changes in the heart that lead to asymptomatic or symptomatic ventricular dysfunctions. Here, significant histological changes were seen between groups: first, DOXO treatment increased cardiac fibrosis, hypertrophy, balloon and necrosis (Figure 7, central figures) compared to the control group. These results agree with other previous studies from our research group. Second, treatment with Singo alone did not change the cardiac histological structure compared to the control group, neither increasing fibrosis, hypertrophy nor necrosis, confirming that it was safe in the heart after oral administration in mice (Figure 7, figures on the left). Notably, the pre-treatment with Singo for 3 days and the subsequent 7 days of combinatorial treatment between DOXO and Singo significantly improved the cardiac histological picture, reducing cardiac fibrosis, hypertrophy, the number of observable necrotic cells and the presence of balloon (associated with a reduction in myocardial inflammation) (Figure 7, figures on the right).

**Figure 8:**
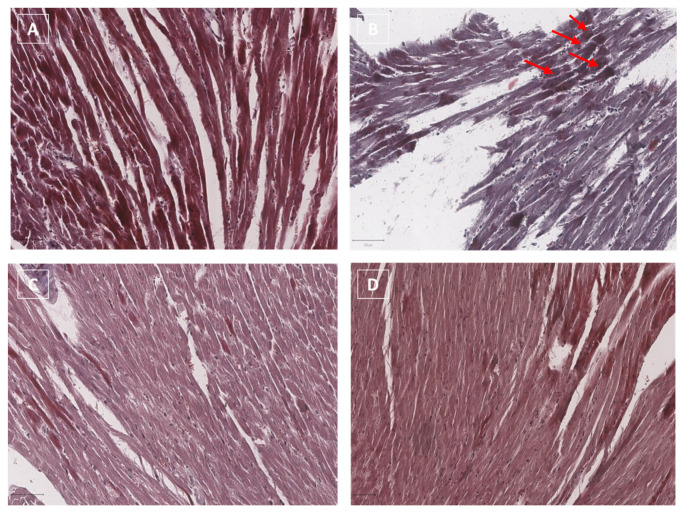
Masson’s trichrome staining in myocardial tissues of mice untreated (saline), (**A**) treated with DOXO (**B**) or Singo (**C**) or both in combination (**D**). Scale bar: 20 µm. Red arrows indicate the points of greatest fibrosis induced by the treatment with DOXO.

Masson’s Trichrome is a commonly used collagen staining method for cardiovascular fibrosis detection. Masson’s trichrome staining in myocardial tissues (Figure 8) clearly indicates that DOXO increases cardiac fibrosis (see red arrows) compared to untreated mice (Figure 8B vs. Figure 8A, respectively). The staining of the collagen fibers can be highlighted in blue using Masson’s trichrome method; in this case, in Figure 8B, the collagen fibers can be detected in a color close to blue due to the overlap with the red/pink corresponding to the cytoplasm staining of cardiac cells. Collagen fibers in the cardiac tissue of Singo mice are not detectable through Masson’s trichrome staining, (Figure 8C). Moreover, in DOXO–Singo mice, collagen staining in myocardial tissues is almost completely absent, confirming the anti-fibrotic activity of Singo during anthracycline treatment (Figure 8D). Comparing the tissue structure of DOXO (Figure 8B) and DOXO–Singo (Figure 8D), cardiac muscle cells with a more linear and homogeneous parallel band structure can be seen in the DOXO–Singo group compared to the DOXO mice (where the tissue structure is more disorganized, with a non-linear shape of the cardiac muscle cells).

### 3.6. Terminal dUTP Nick End-Labeling (TUNEL) Assay

**Figure 9:**
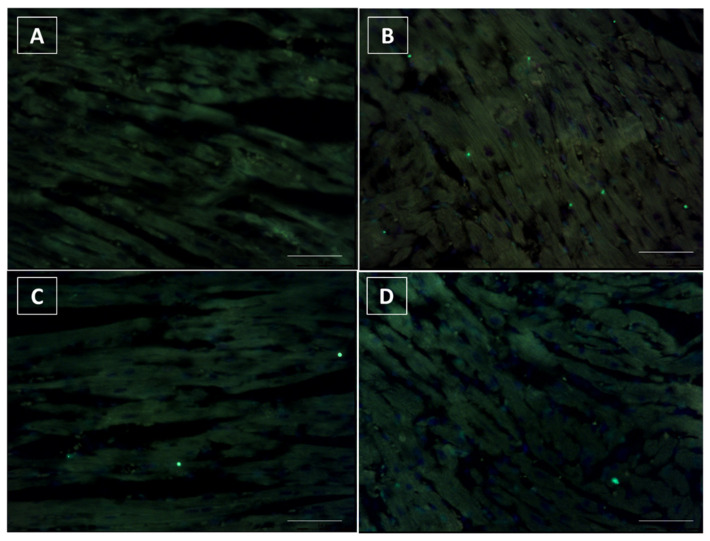
TUNEL assay indicating the fragmented DNA in tissue samples of untreated mice (saline) (**A**) or those treated with DOXO (**B**), Singo (**C**) or both in combination (**D**). Scale bar: 20 µm.

To test whether Singo treatment inhibits DOXO-induced cardiac in apoptosis in vivo, we examined heart sections by TUNEL assay. TUNEL-positive cardiomyocytes were barely detectable in the heart of untreated mice (Figure 9A). A similar picture was seen in Singo-treated mice (Figure 9B), where only rare spots of apoptotic cells were seen. Notably, DOXO therapy significantly increased the number of TUNEL-positive cardiomyocytes (Figure 9B), indicating a pro-apoptotic phenotype induced by anthracycline treatment. Conversely, this change was markedly attenuated in the DOXO–Singo mice (Figure 9D), indicating the anti-apoptotic properties of Singo in preclinical models.

### 3.7. Cell Viability

**Figure 10:**
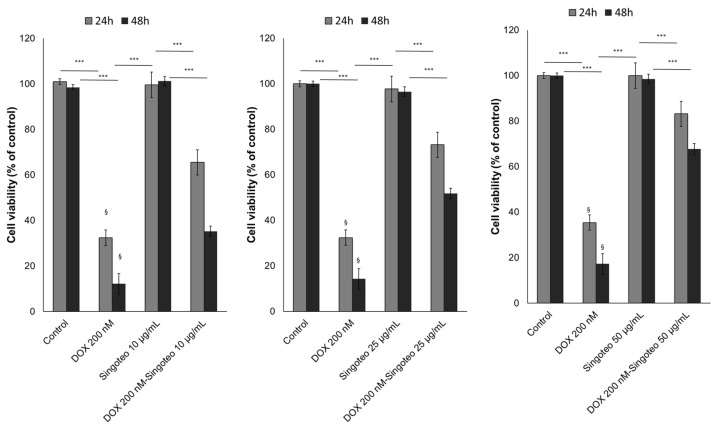
Cell viability of human cardiomyocytes exposed to DOXO at 200 nM, Singo at 10, 25 and 50 µg/mL, or both in combination for 24 and 48 h. Results: mean +/− SD. *** *p* < 0.001. §: *p* < 0.001 between the DOXO 200 nM group and DOXO 200 nM associated with Singo 10 µg/mL.

A time- and concentration-dependent cardioprotective effect of Singo was seen in human cardiomyocytes exposed to DOXO at subclinical concentrations. As indicated in another recent publication [57], DOXO at 200 nM reduces the cell viability of cardiomyocytes by 65–70 and 90–95% after 24 and 48 h. Growing Singo concentration from 10 to 50 µg/mL co-incubated with DOXO significantly reduced the magnitude of the effects (Figure 10).

### 3.8. Lipid Peroxidation

**Figure 11:**
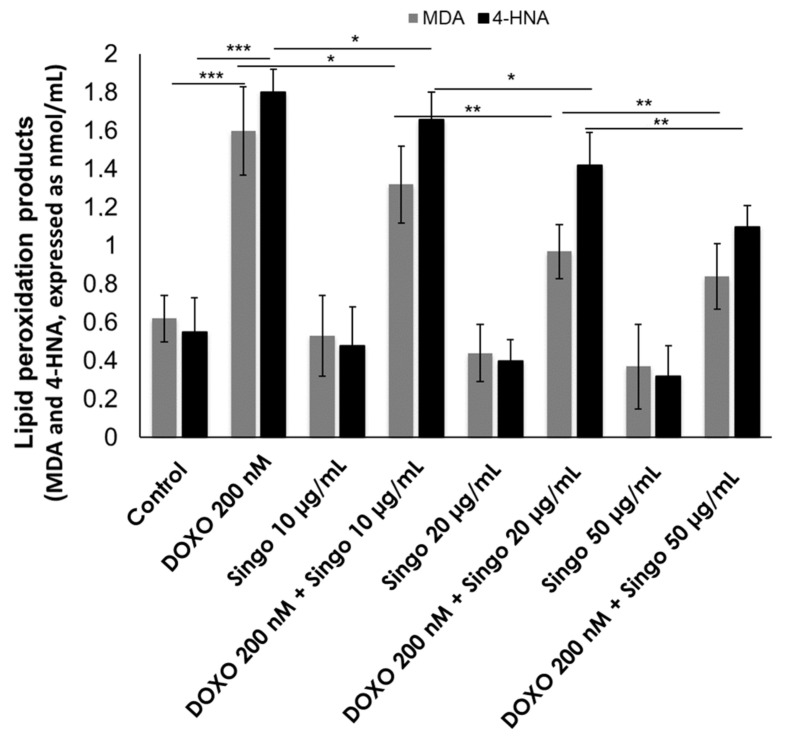
Lipid peroxidation products (MDA and 4HNA) expressed as nmol/mL were quantified in human cardiac cells unexposed (control) or exposed to DOXO (200 nM), Singo (10, 25 and 50 µg/mL) or both in combination for 24 h. Results: mean +/− SD. *** *p* < 0.001. ** *p* < 0.01. * *p* < 0.05.

DOXO exerts cardiotoxic effects through the induction of ferroptosis and lipid peroxidation products (MDA and 4HNA) that induce cell damage and apoptosis [58]. Nutraceuticals are able to reduce ROS formation and lipid peroxidation processes as indicated in the literature [59]. Here, Singo demonstrated abilities to reduce both MDA and 4-HNA levels in human cardiomyocytes exposed to DOXO (Figure 11). These effects are concentration-dependent and demonstrate high anti-oxidative properties of the nutraceutical formulation.

### 3.9. Inflammasome and p65/NF-kB Expression in Human Cardiomyocytes

**Figure 12:**
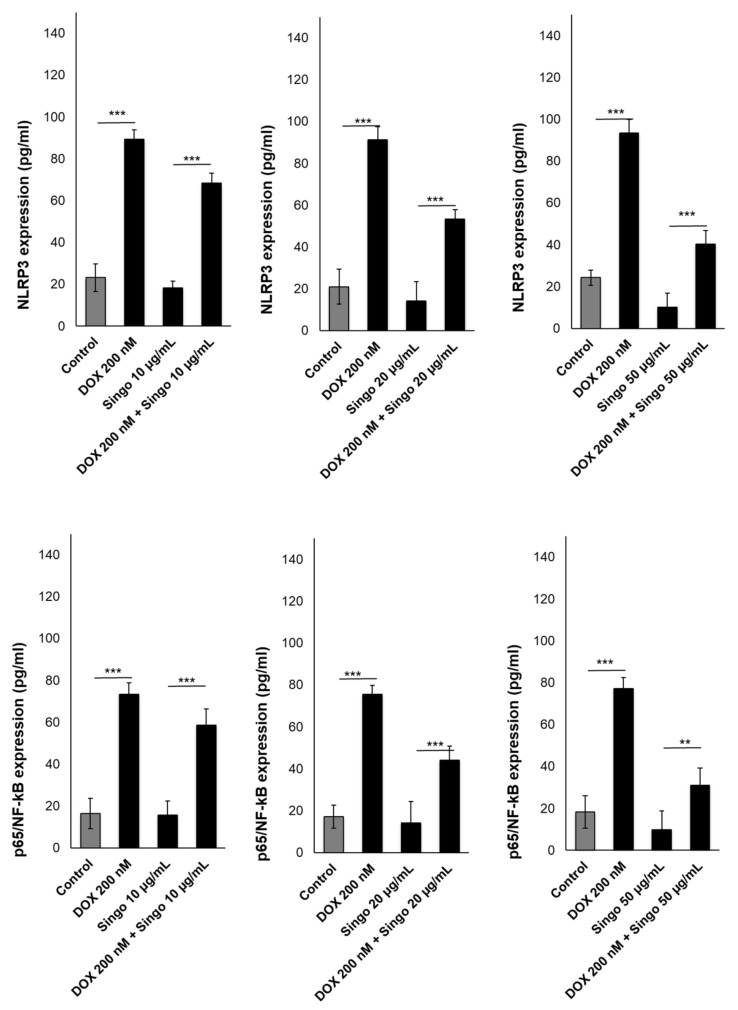
NLRP-3 inflammasome (upper panels) or p65/NF-kB (lower panels) expressed as pg/mL were quantified in human cardiac cells unexposed (control) or exposed to DOXO (200 nM), Singo (10, 25 and 50 µg/mL) or both in combination for 24 h. Results: mean +/− SD. *** *p* < 0.001. ** *p* < 0.01.

The activation of the NLRP3/NF-kB pathway is a key orchestrator of anticancer-induced cardiotoxicity. This pathway activates pro-inflammatory cytokines and hs-CRP levels in patients with CVD [60]. In human cardiomyocytes exposed to DOXO, the NLRP3 and p65/NF-kB levels were strongly enhanced compared to untreated cells (Figure 12). When co-incubated with Singo, their concentration changes significantly. In fact, the NLRP3 levels (pg/mL) were reduced by 20%, 40% and 60% in cells exposed to Singo at 10, 15 and 50 µg/mL, respectively, compared to only DOXO-treated cells after 24 h. The same picture was seen in the p65/NF-kB intracellular levels.

#### Cytokinome Profile Evaluation in Human Cardiomyocytes

**Figure 13:**
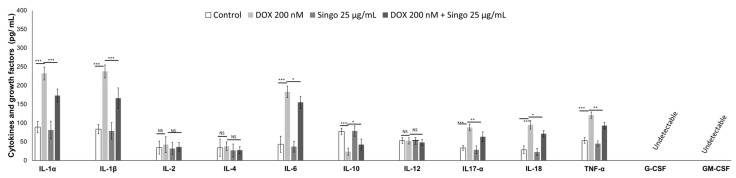
Cytokines and growth factors (IL-1α, IL-1β, IL-2, IL-4, IL-6, IL-10, IL-12, IL-17α, IL-18, IFN-γ, TNF-α, G-CSF and GM-CSF) involved in cardiotoxicity and inflammation expressed as pg/mL were quantified in human cardiac cells unexposed (control) or exposed to DOXO (200 nM) or Singo (25 µg/mL) or both in combination for 24 h. Results: mean +/− SD. ns: not significant. *** *p* < 0.001. ** *p* < 0.01. * *p* < 0.05.

A pro-inflammatory cytokinome profile in cancer patients treated with anticancer drugs is strongly associated with cardiomyopathies and reduced overall survival [61]. In human cardiomyocytes exposed to DOXO, the IL1αβ, IL-6, IL-17α, IL-18 and TNF-α levels were strongly enhanced compared to untreated cells (Figure 13). When co-incubated with Singo, the cytokine levels were significantly reduced in cells exposed to Singo at 25 µg/mL compared to only DOXO-treated cells after 24 h. An opposite effect was seen in the intracellular IL-10 level in cells co-incubated with Singo and DOXO compared to cells treated with DOXO alone (Figure 13).

## 4. Discussion

Cancer remains a leading cause of death determining more than 10 million deaths in the last two years [62]. However, due to pharmacological improvements, cancer-related mortality is still reducing due to standard chemotherapies, including anthracyclines, targeted therapies and immune checkpoint inhibitors [63]. The distinct mechanisms of anticancer therapies are often associated with adverse effects, including cardiotoxicity, such as chemotherapy-associated cardiomyopathy (CAC) [64]. Some standard chemotherapy regimens involve anthracyclines alone or combined with targeted therapies [65]. Doxorubicin-induced cardiotoxicity remains a key side effect seen both in younger and older cancer patients [66]. The mechanisms of DOXO-induced cardiotoxicity involve oxidative stress, lipid peroxisomes, interleukin-1 overexpression, NLRP-type 3 activation and mitochondrial dysfunctions in cardiac cells [67]. Novel potential cardioprotective strategies, including anti-inflammatory and anti-fibrotic nutraceuticals or glucose- and cholesterol-targeting drugs, should be analyzed in preclinical models of cardiotoxicity in order to reduce myocardial injuries through an in-depth analysis of the biochemical mechanisms of cardioprotection [68]. Inflammation plays a key role in both cancer cell survival and cardiotoxicity phenomena [69]. Myocardial inflammasome and systemic cytokine levels correlate with cardiovascular diseases and anticancer-induced cardiotoxicity events in patients with cancer [70]. Actually, the appropriate management of DOXO doses and the use of cardiology drugs generally are well tolerated and in some cases improve cardiac function in patients with cancer; however, more translational research is needed to reduce cardiomyopathies, heart failure, heart fibrosis and inflammation [71]. Complementary and alternative medicine (CAM) involves nutraceuticals with antioxidant, anti-inflammatory and cardioprotective properties [72,73,74]. Here, a combination of *Spirulina platensis*, *Ganoderma lucidum* and *Moringa oleifera*, called Singo, was successfully tested for DOXO cardiotoxicity in preclinical models. These natural bioactives are well known in the clinical management of cancer-related fatigue and pain and for improving quality of life [75].

It is well known that anthracyclines cause decreased cardiac function (mainly seen as EF and longitudinal and radial strain). These functional effects are the result of a series of pro-inflammatory and pro-fibrotic biochemical events leading to cardiac apoptosis, ferroptosis and a loss of the metabolic balance of the cardiomyocyte. Notably, pre-treatment with Singo for three days and subsequent co-administration with DOXO resulted in a significant increase in the ejection fraction, left ventricular ejection fraction and radial/longitudinal strain in mice (Figure 4). In fact, these may be the result of both systemic and cardiac anti-inflammatory effects induced by the nutraceutical, including the reduction in both the systemic and tissue levels of the cytokines involved in cardiotoxicity. It has been established that IL-1 and NLRP-3 inflammasomes are key drivers of anticancer drug-mediated cardiac dysfunctions, including heart failure, a decrease in the left ventricular ejection fraction, EF and cardiac fibrosis [76,77]. Therefore, we investigated the expression of IL1, IL6 and NLRP3 in cardiac tissues of mice treated with DOXO alone or combined with Singo. DOXO increased IL-1, IL-6 and NLRP-3; contrariwise, Singo did not change their expression in the myocardial levels after oral administration, indicating a safety profile in preclinical models. Notably, DOXO–Singo mice showed a significant reduction in the expression of cardiotoxicity biomarkers, indicating a beneficial effect on cardiac inflammation, fibrosis and apoptosis. Histological analyses in hematoxylin and eosin and trichrome confirmed that anthracyclines induce heart damage and that nutraceutical treatment has a preventive effect on cardiac hypertrophy, necrosis, apoptosis and fibrosis. Specifically, a notable increase in cardiac fibrosis, hypertrophy and necrosis was seen in the DOXO mice vs. the untreated mice (Figure 9 and Figure 10). In contrast, in the DOXO–Singo mice, a totally different behavior was seen, with a marked improvement of the histological picture, resulting in less cardiac hypertrophy, necrosis and fibrosis.

Anthracyclines induce cardiac apoptosis even after a few days of treatment in preclinical models [36,43]. TUNEL analyses confirmed that DOXO dramatically increases TUNEL-positive cardiomyocytes, while treatment with Singo significantly reduces their number. However, this study has several limitations: first, we used non-cancer bearing mice during the DOXO therapy; this cancer-free model could exclude potential differences due to hormonal and immune changes due to cancer progression [78]. However, this preclinical model is often used to study potential new cardioprotective drugs in cardio-oncology [79,80], including nutraceuticals. Another limitation is the relatively low anthracyclines exposure in this study; however, the short-term cardiotoxic and pro-inflammatory effects of DOXO are already extensively described in the literature on both human and mouse models [81], thus providing scientific information on the short-term cardiac toxicity of anthracyclines and possible natural strategies in primary prevention. For this purpose, the cardioprotective and anti-inflammatory effect of Singo could be considered a potential strategy in the primary prevention of cardiac dysfunctions induced by anthracyclines. Further studies on the antitumor efficacy of DOXO associated with Singo will be performed in preclinical models. Moreover, another limitation of the study is the need for a deeper proteomic study based on the effects of Singo on DAMPs and inflammasome-related pathways through Western blot and metabolomic methods. A more appropriate proteomic profile on cardiac cells exposed to DOXO and Singo will be analyzed in subsequent studies. The overall picture of this preclinical study indicates a new potential cardioprotective strategy against anthracycline-mediated cardiotoxicity. Singo has shown beneficial effects on both histological and biochemical levels by preventing a reduction in cardiac function parameters in mice after oral administration. Randomized-controlled trials in breast cancer patients treated with anthracyclines alone or in combination with Singo are being planned at the Istituto Nazionale Tumori-IRCCS-Fondazione G. Pascale of Naples.

## 5. Conclusions

A nutraceutical combination *of Spirulina platensis, Ganoderma lucidum* and *Moringa oleifera* is able to reduce inflammation and myocardial dysfunctions induced by DOXO in preclinical models. The putative pathways of nutraceuticals-induced cardioprotection involve the inflammasome and pro-necrotic and pro-fibrotic cytokines related to reductions in the ejection fraction and radial/longitudinal strain. This preclinical study confers the first scientific evidence of cardiovascular benefits induced by complementary and alternative medicines (CAM) in cardio-oncology. Clinical studies in cancer patients treated with DOXO and supplemented with a combination of *Spirulina platensis, Ganoderma lucidum* and *Moringa oleifera* should be considered.

## Data Availability

Raw data are available at this Zenodo link: https://zenodo.org/record/7043090#.YxHJN4rP1D8 (accessed on 2 September 2022).

## Supplementary Materials

The following supporting information can be downloaded at: https://www.mdpi.com/article/10.3390/jcdd9120423/s1.
